# Student physiotherapists perceptions of online curriculum delivery during the COVID-19 pandemic

**DOI:** 10.1186/s12909-022-03486-5

**Published:** 2022-06-07

**Authors:** Paul Chesterton, Mark Richardson, Craig Tears

**Affiliations:** School of Health and Life Sciences, Teesside University, Borough Road, Tees Valley, Middlesbrough, TS1 3BA UK

**Keywords:** COVID-19, Online learning, Health education, Students, Satisfaction

## Abstract

**Background:**

As a result of the COVID-19 pandemic a rapid transformation from face-to-face curriculum delivery to an online teaching and learning environment, was adopted in a number of higher education institutions globally. Allied Health Profession courses such as physiotherapy, traditionally utilising an in person teaching model to prepare students for practice, needed to swiftly adopt new methods of delivery, involving both synchronous and asynchronous approaches. Understanding physiotherapy student perceptions of this transition is important to allow faculty to develop their delivery of online teaching and provide an evidence base for future course curricula.

**Methods:**

Cross-sectional survey of UK higher education students studying either an undergraduate or post-graduate pre-registration degree in physiotherapy was conducted between October 2020 and February 2021. The survey investigated the student’s perception of the transition to either an online or hybrid model of learning during the COVID-19 pandemic. A mixed method approach was adopted allowing respondents to share their experiences and facilitate the exploration of questions which required in-depth thought.

**Results:**

Two hundred thirty-six respondents completed the questionnaire. Online learning was perceived to be a flexible (49%, *n*=116,CI 95% 43 to 55) and convenient (49, 116, 43 to 55) method of learning. Despite this, 79% of the students surveyed felt that the online learning experience had a negative impact on their understanding of the subject and were disadvantaged compared to traditional face-to-face teaching provision (mean 4.14 ± SD 1.06). Online physiotherapy delivery produced low student satisfaction, leaving respondents feeling disadvantaged. Decreased levels of engagement and the lack of ability to practice ‘hands-on’ skills were detrimental aspects of the online approach, with 55% (*n*=106) reporting they did not perceive the academic staff had the necessary skills to deliver effective online content.

**Conclusions:**

The majority of UK physiotherapy students surveyed were dissatisfied and lacked engagement with an online learning approach within the curricula, compared with the traditional face-to-face delivery. Although several positives of both a synchronous and asynchronous delivery were highlighted, faculty must consider how they best deliver online learning content, making use of pedagogical strategies that will create as many learning and engagement opportunities as possible.

**Supplementary Information:**

The online version contains supplementary material available at 10.1186/s12909-022-03486-5.

## Background

A novel coronavirus, COVID-19 was discovered in December 2019 [1]. Clinical analysis of the virus demonstrated that transmission between persons was increased through close contact and social distancing was announced as an early strategy to reduce disease transmission [2–4]. The World Health Organisation (WHO) declared COVID-19 as a global public health emergency of international concern on 30th January 2020, and then officially a pandemic on 11th March 2020 [5]. The first UK cases were reported on 31st January 2020 and a national lockdown was declared on March 23rd 2020. Within the United Kingdoms (UK) Higher Education community all university campuses closed, and courses forced to rapidly transition from a face-to-face to online learning environment. Many institutions have therefore become interested in how to best deliver the course content online, engage learners and conduct assessments [6].

The UK physiotherapy curriculum adopts a blended delivery strategy, traditionally from an on-campus presence provided by an academic faculty and placement education located within clinical practice. The academic faculty provide a major part of fundamental physiotherapy teaching in a physical classroom environment with the assessment of the theoretical and practical components of the curriculum [7]. Clinical placements have been considered the most influential method of learning which prepared physiotherapy students for employment within the UK [8]. Chesterton and colleagues [8] reported that within the academic environment practical seminars were the most valued, preparing UK physiotherapy students for graduation and clinical practice. Online learning was the least preferred method but reasons were not explored and with the recent global pandemic forcing, at least in part, the physiotherapy curriculum online this is a key aspect of future facing education which requires investigation [8]. Online learning can be highly effective in digitally advanced countries [9]. Hrastinski [10] stated there were two types of online learning: synchronous and asynchronous and to be effective and efficient, lecturers and institutions must have a comprehensive understanding of the advantages and disadvantages of each. Synchronous learning involves students learning together in live environments, which allows greater engagement and sense of community [11]. Conversely, asynchronous learning allows students to learn material individually in a flexible way providing time for content synthesis [12].

Opportunities such as increased flexibility, interactivity, self-pacing, comfort and accessibility have all been highlighted as advantages of online learning, [6, 13–18] with a mix of synchronous and asynchronous material purposed to be effective [19]. However, Branch and Dousay [20] suggest that effective online learning is a by-product of cautious design and planning, neither of which were possible in the COVID-19 pandemic where rapid transformation to online learning was required.

The benefit of online learning in some non-clinical topics has been highlighted previously [21]. There is limited understanding of the impact of online learning for clinical skills. In a recent systematic review, it was argued that online learning for undergraduate health professions was equivalent and possibly even superior to traditional methods of curriculum delivery [22]. The high risk of bias among several included studies, however, precluded the authors from drawing definitive conclusions. In comparison, others have challenged the compatibility of online learning with courses where hands on practical experiences are required as part of instructional activities [15]. Furthermore, students have also expressed concerns that remote learning impacted their ability to develop clinical competence, [23] and were fearful about potential employer discrimination against those who study through e-learning [24]. The literature investigating student perceptions of online learning for clinical skills has been generally conducted with medical students, with little known regarding student perceptions in the allied health professions including physiotherapy.

No study to the authors knowledge investigating UK physiotherapy student’s perceptions of online learning during COVID-19 has been conducted, and thus our study aims to be the first to explore this. With the increase in use of digital learning during COVID-19, it is necessary to understand its effectiveness with regards to learning and teaching from various stakeholders. Student perceptions are essential regarding the advantages, limitations, and recommendations for online learning. Reinholz & French [25] have also suggested that digital health platforms for both patients and students will remain an integral part of care even after the COVID-19 pandemic. Thus, our exploratory study aims to provide a greater understanding of the perceived benefits and limitations which will allow allied health professions, such as physiotherapy to develop their delivery of online teaching and provide an evidence base for future course curricula. This study also aims to identify constructs to generate hypotheses for future research to develop the evidence base behind this method of curriculum delivery.

## Methods

### Design

A cross-sectional exploratory survey of UK higher education students studying either an undergraduate or post-graduate degree in physiotherapy (pre-registration) was conducted between October 2020 and February 2021. The School of Health and Life Sciences Ethics Committee at Teesside University approved the study in accordance with the Helsinki Declaration (ID1695).

The questionnaire aimed to investigate the student’s perception of the transition to either an online or hybrid model of learning during the COVID-19 pandemic. Therefore, a mixed method approach was adopted allowing respondents to share their experiences and facilitate the exploration of questions which required in-depth thought [26]. The questions were designed to develop an understanding of the student attitudes, experience and satisfaction with the transition to online learning. Quantitative questions were designed for an online format and included dichotomous, multiple choice or Likert scale, of which all scales were unipolar. The Likert scale questions were scored as follows: 1, Strongly Disagree; 2, Disagree; 3, Impartial; 4, Agree; 5, Strongly Agree. Qualitative questions were open ended, through the use of open text boxes, to capture students’ reflections in greater detail. In addition to quantitative questions, participants were given the option to expand on answers where appropriate. The qualitative questions (Q21, Q22, Q23) were included to provide a richness of respondent’s thoughts and experiences, to determine and provide additional understanding and meaning behind the dataset. This was employed using thematic analysis within the work of Braun and Clarke [27]. This method, based on an inductive approach, can reveal unanticipated insights which may provide further richness to the dataset [28].

The survey was initially piloted and assessed for content validity by six physiotherapy lecturers [29]. Academics involved in the pilot independently assessed the survey providing comments on the format, content, wording and overall ease of completion. Following the removal of (two) and rewording of (five) questions, the survey was further piloted by 12 physiotherapy students (8 Bachelor of Science (BSc), 4 Master of Science (MSc)). Following a further round of piloting the final online survey (Onlinesurveys.ac.uk) consisted of a total of 23 main questions of which some included further sub-questions (Supplementary Material 1). The first section of the survey captured initial participant background characteristics including age, gender, ethnicity, type of degree and year of study. The second section of the survey asked a range of questions related to online curricula delivery and depending on the answers provided participants were re-routed through the survey. No data which could identify personal participants including University studied at was collected to maintain anonymity.

### Participants

All students who were currently enrolled on an undergraduate or a post-graduate pre-registration UK physiotherapy programme at the time of investigation were eligible to take part in the survey. The survey was distributed via the Chartered Society of Physiotherapy interactive website (iCSP https://www.csp.org.uk/icsp) as a means of capturing student members from across the UK. A snowball sampling technique was also employed to facilitate the distribution of the survey within higher education settings [30]. Respondents were instructed that by completing and submitting the survey they were consenting to take part.

### Statistical analysis

The data were extracted from onlinesurveys.ac.uk into Microsoft Excel (Microsoft Corp, Redmond, WA) using the analyse function following survey closure. The survey was not designed to test for differences between respondents and, therefore, no such analysis was performed. Presented is descriptive data with results from the dichotomous questions converted into proportions with lower and upper limits of the 95% confidence interval [31]. Likert-scale questions were treated as numeric variables [32]. The mean and SD were calculated for combined responses across each potential answer.

The open-ended answers provide a more ecologically valid response but created an extra stage in analysis. Qualitative responses were exported into a document to allow familiarisation of the respondent’s comments to be reviewed by P.C [27]. Similar comments were coded manually and enabled the researcher to group potential broad categories. These categories were further reviewed where additional coding permitted a set of sub and main themes to be created. These themes were redefined and re-grouped if advised following a triangulation and peer debriefing process involving another researcher (M.R). For any disagreements (e.g. formation of themes, coding) a third researcher (C.T) would provide an independent review of the disagreement, however, no such disagreements occurred. Hermeneutic revisiting of the data set reduced researcher prejudices or biases which may have de-valued the theme generation. Comments that did not align to any of the themes and deemed not to provide additional insights to the phenomenon were discarded.

## Results

### Participant demographics

Two hundred thirty-six respondents completed the questionnaire and were included in the analysis. Ages ranged from 18 to 56 (Mean **±** SD, 25.5 + 8.0) with 78 identifying as male (33%), 156 female (66%) and two as self-identifying other (1%). 163 respondents were enrolled on a current BSc programme (69%) with the remaining 73 (31%) MSc students. Years of study included 1 (*n*=68, 29%), 2 (92, 39), 3 (71, 30) and 4 (5, 2).

### Transition to online learning

Respondents were asked how they found the transition to online learning from face-to-face delivery. In total 51% (*n*=119, 95% CI 44 to 57) were ‘apprehensive about the change’, 33% (78, 27 to 39) ‘could see the benefits/opportunities of using technology’ and 10% (24, 7 to 15) were ‘not worried/concerned’ with the transition. Of those who answered ‘Other’ (6%, 15, 4 to 10), respondents found the transition stressful and difficult with a perception of missing out on clinical skills. 159 respondents (67%) either ‘strongly disagreed’ (32%, 76, 27 to 39) or disagreed (35%, 83, 30 to 42) that they were equally motivated to learn using an online platform compared to a face-to-face traditional approach. The lack of daily structure, distractions during sessions, lack of cohort interaction and ability to practice hands-on skills were reasons cited.

Respondents were asked questions in relation to their satisfaction of the online learning approach of their pre-registration degree programme, whether they felt disadvantaged with the online approach and the support received from faculty Table 1.

**Table 1:** Respondents views of online learning

How much do you agree with the following:	Combined respondent answer (mean ± SD)
I feel at a disadvantage with online compared to face-to-face learning	**Agree** (4.14 ± 1.06)
I am satisfied with the online learning approach within my degree programme	**Impartial** (2.76 ± 1.13)
I feel equally motivated to learn using an online learning compared to a face-to-face approach	**Disagree** (2.22 ± 1.15)
I have felt supported by my tutors for online learning sessions	**Impartial** (3.13 ± 1.18)

Likert Scales: 1, Strongly Disagree; 2, Disagree; 3, Impartial; 4, Agree; 5 Strongly Agree

The opportunities to engage in online classes were not considered the same as face-to-face (63%, *n*=149, 95% CI 57 to 69). Only 45 respondents (19%, 15 to 25), felt the opportunities to engage were the same as face-to-face classes. In addition to engagement, just over half of respondents (55%, *n*=106) felt academic staff and lecturers had the necessary skills to deliver effective online content. Respondents experienced a wide variety of teaching practices, similar to the diversity experienced in face-to-face classes, however identified that academic staff may not be technologically proficient or confident to provide an innovative learning experience. This inconsistency was a theme of the respondents; however, several acknowledged that the transition to online delivery was challenging for faculty members.

### Perceived advantages and disadvantages of online learning

Respondents were asked what they perceived as both the advantages Table 2 and disadvantages Table 3 to online learning.

**Table 2:** Perceived advantages of online learning

Available Answer	Respondents N (%, CI)
Improve use of technology and digital skills for future use	137 (58, 52 to 64)
Interact with groups of students virtually	54 (23, 18 to 29)
More confident to interact/answer questions (secondary to anonymity)	57 (24, 19 to 30)
Find digital quizzes useful for learning	73 (31, 25 to 37)
Find video’s/demonstrations useful to supplement learning	85 (36, 30 to 42)
Able to learn at own pace better (secondary to recorded sessions)	116 (49, 43 to 55)
The convenience of learning in home environment	116 (49, 43 to 55)
Other	7 (3, 1 to 6)
I don't believe there are any advantages to Online learning	24 (10, 6 to 15)

**Table 3:** Perceived disadvantages of online learning

Available Answer	Respondents N (%, CI)
Lack of cohort identify	168 (71, 65 to 77)
Unable to develop close relationships with peers	194 (82, 77 to 87)
Lack of peer feedback during sessions	175 (74, 68 to 87)
Lack of one-to-one feedback from tutors	170 (72, 66 to 77)
Unable to practice ‘hands on’ skills	222 (94, 90 to 96)
Decrease chances of employability – lack exposure to clinical skills	149 (63, 57 to 69)
Lack of confidence in using the technology	40 (17, 13 to 22)
Connectivity issues during sessions	149 (63, 57 to 69)
Pace of delivery can be affected	127 (54, 47 to 60)
Doesn’t meet preferred learning style	125 (54, 47 to 59)
Lack of designated workspace or study area (either at University or personal residence)	118 (50, 44 to 56)
Other	9 (4, 2 to 7)
I don't believe there are any disadvantages to Online learning	0

In addition, to the options available, students identified the often-shorter sessions were time efficient. The convenience of speaking to tutors and the safety of the home environment during the pandemic were also considered advantages of an online learning delivery.

A total of 186 respondents (79%) felt online learning had a negative impact on their understanding of the subject area. The primary reason for this was a lack of confidence in applying clinical and practice skills (163, 88%, 82 to 92), followed by connectivity issues experience during classes (71, 38%, 31 to 45) and the pace of session delivery (70, 37%, 31 to 45). To improve the online experience and facilitate high quality education respondents felt institutions should provide dedicated online learning spaces (54, 128, 48 to 60), provide the necessary hardware (38, 89, 32 to 44) and provide detailed explanations of the benefits of the online method (26, 61, 21 to 32). For the 50 respondents who felt online learning had not impacted negatively on their learning experience, the use of technology (33, 66%, 52 to 78) and availability of recorded sessions (28, 56%, 42 to 69) had allowed for better subject understanding.

### Synchronous and asynchronous delivery

Respondents in the main preferred a mixture of synchronous and asynchronous online delivery (60%, 142, 54 to 66). Table 4 displays respondents perceived benefits of synchronous online delivery. Respondents also suggested that live feedback from questions posed to lecturers was considered advantageous.

**Table 4:** Perceived benefits of synchronous delivery

Available Answer	Respondents N (%, CI)
Can clarify points with tutors	205 (87, 82 to 91)
More interaction with peers	156 (66, 60 to 72)
Structured learning (makes you attend the session)	165 (70, 64 to 75)
More engaged with subject material	142 (60, 54 to 66)
More motivated to learn	109 (46, 40 to 53)
Other	5 (2, 1 to 5)
I don't believe there are benefits to synchronous learning	2 (1, 0 to 3)

Table 5 includes the benefits of asynchronous delivery with respondents suggesting that the ability to stop presentations, rewind, and repeat the explanation of subject specific content were advantageous for their learning.

**Table 5:** Perceived benefits of asynchronous delivery

Available Answer	Respondents N (%, CI)
Learn at your own pace	175 (74, 68 to 79)
Watch recordings as many times as you wish	189 (80, 75 to 85)
Flexible learning (i.e. at a time of your choice)	179 (76, 70 to 81)
Improves work/life balance	95 (40, 34 to 47)
Other	3 (1, 0 to 4)
I don't believe there are benefits to asynchronous learning	10 (4, 2 to 8)

Respondents were asked what they would find helpful for their subject understanding in both synchronous and asynchronous sessions. The themes generated from this question are displayed in Tables 6 and 7.

**Table 6:** Themes of synchronous sessions which would support student learning

**Theme: Additional interaction**	
Varied interaction	‘Would be really useful to have a wider range of interactive opportunities. This would increase engagement and interest within the session, especially when watching from home and all the distractions that brings.’ ‘The ability to anonymously message the lecturer to ask questions or have discussions would consolidate understanding.’
Linked to clinical scenarios	‘More thought-provoking discussions needed, with patients involved in discussions as we have on campus’. ‘Greater application to clinical scenarios and case studies, which is even more important when delivered online’.
Personal approach	‘Cameras are sometimes all off to allow for better signal, and this can make the session hard to understand who’s speaking and feels less personal.’ ‘No one turns videos on or microphones half the time. Therefore, we are just left looking at slides being talked over, and you feel less invested in the session.’
**Theme: Smaller groups**	
Encourages engagement Group discussion	‘Smaller groups work better, I feel this gives me the opportunity to engage more.’ ‘Less students would allow more group work, and increase the transferability to campus teaching… which needed linking up more.’ ‘If we had less people in the sessions it would provide more opportunities for group discussion. We have had this at times, but with large groups you can’t always participate, and it leaves some students unable to contribute.’
**Theme: Teaching techniques**	
Diverse teaching techniques	‘More tasks to do during the live sessions such as quizzes, word clouds, polls, break out groups.’ ‘Each online lesson becomes the same very quickly without variety in delivery methods.’ ‘Opportunities to interact, I don’t like the sound of my own voice, but it’s much more beneficial to speak rather than type.’
Overreliance on supporting content	‘A variety of presentation methods needed especially when studying online most of the day.’ ‘More opportunities to interact and learn in different ways. Death by PowerPoint.’

**Table 7:** Themes of asynchronous sessions which would support student learning

**Theme: Pre-reading materials**	
Earlier access to pre-reading materials	‘It would be useful to view materials related to teaching earlier to provide us with an opportunity to read/digest information.’ ‘Pre-recorded materials to be recorded in “chunks” rather than whole sessions and not just recorded live sessions from previous years would be most useful’. ‘Earlier access the better as it’s difficult to always follow-up with suggested material after the lecture when you’re moving onto the next topic.’
Variety of pre-reading resources	‘I enjoyed listening to the pre-records more like podcasts, so being able to separately review slides later was beneficial (i.e., without having to review a 50min video recording to see the slides).’ ‘Links to associated articles prior to the lectures helped with overall understanding.’
**Theme: Academic Input**	
Follow-up discussion with academic faculty	‘Reflection / pre-post evaluation of learning objectives with staff to show understanding gained through session would be beneficial.’ ‘Attaching a Padlet or something similar so that any further questions could be addressed with the lecturer.’
Additional support sessions	‘Providing a forum for questions rather than having to email lecturers individually for answers.’ ‘Additional one-offs ask the lecturer events or more structured additional touch points needed to ensure a similar experience as face-to-face teaching.’ ‘Lecturers often difficult to contact so structured question and answer sessions immediately following timetabled recorded sessions would provide a structure and an opportunity to gain understanding.’
**Theme: Learning validation**	
Interactive approaches to post session understanding	‘It would supplement our learning if we were given short quizzes on what was just discussed to make sure we understood the key points.’ ‘Possibly an exit quiz or discussion board at the end of each session to make sure I’ve appreciated the key take home messages.’
Variety of learning opportunities	‘Session content should be reinforced in following synchronous sessions.’ ‘Having a variety of post session engagement tasks including summary slides/question boards monitored by tutors would provide me with confidence in my understanding.’

## Discussion

Our study is the first to explore UK physiotherapy students’ perceptions of the online learning delivery during COVID-19 pandemic. Our novel and key findings include that overall students’ surveyed (79%) felt that the online learning delivery had a negative impact on their understanding of the subject and were disadvantaged compared to face-to-face traditional provision (mean 4.14 ± SD 1.06). Online physiotherapy delivery produced low student satisfaction, leaving respondents feeling disadvantaged by the model with decreased levels of engagement and the ability to practice ‘hands-on’ skills, a cornerstone of the profession. Despite this, physiotherapy students did highlight some advantages to online learning and preferred a mixture of synchronous and asynchronous approaches to delivery, which should be considered when designing pedagogical strategies to create as many learning and engagement opportunities as possible.

### Transition to online learning

Over half of the physiotherapy students who completed the survey were apprehensive about the transition to online learning. This was reinforced by qualitative comments reflecting that the pedological delivery change may impact students’ ability to practice clinical skills.

*‘I have found the transition very stressful, and I’m worried it is going to affect my clinical skills and future employability.’*

*‘It’s been really difficult, and I feel I’m missing out on a LOT of hands-on experience that is vital to our course.’*

Similar experiences have been cited in college students who also reported significant challenges in their learning and life conditions due to the necessity of rapid adjustment and the uncertainty brought by the COVID-19 pandemic [33]. University business students have acknowledged these challenges also with a negative perception of online learning [34]. The new digital pedagogy was deemed inferior to the traditional on campus delivery. The shift to an online teaching strategy requires faculty to strive to understand both the technologies associated with e-learning whilst understanding the need to fundamentally change and transform pedagogical approaches to meet the instructional needs of online students [35–39]. A further layer of nuance is created by the practical nature of the physiotherapy discipline, ensuring that educators need to reconceptualise their teaching methods to meet the demands of an online paradigm shift [37].

### Perceived advantages and disadvantages of online learning

In addition, to a sense of disadvantage, respondents were ‘impartial’ (2.76 ± 1.13) when asked if they were satisfied with the online learning approach provided within their degree. Online instruction has previously been considered a less satisfying learning experience for students [40]. A number of perceived disadvantages of online learning were suggested by respondents with decreased cohort identify, lack of peer feedback and the inability to develop close working relationships (Table 3) recognised as drawbacks of the approach. Conventional classroom socialisation has been identified as a missing component of an online delivery strategy, [18] with real time sharing of ideas, knowledge and information partially absent from the digital world [41]. Ke and Xie [42] suggest online curriculums may only allow superficial communication between students and lecturers which can impact upon the way content is absorbed and understood. Many challenges with online learning have been highlighted in studies across the globe, including connectivity issues, availability of the required technology, digital competence, home distractions and reduced student motivation [17, 43, 44]. This was replicated through our study in both quantitative and qualitative responses with a lack of designated workspace (50%, 118, 44 to 56), connectivity issues (63%, 149, 57 to 69) and confidence in using technology (17%, 40, 13 to 22) all acknowledged as barriers to online learning.

*‘I frequently lose internet connection during sessions resulting in me missing key information and discussions, which gets really frustrating.’*

*‘I simply can’t concentrate to the same extent as when I am on campus, due to family at home, it’s hard to focus.’*

*‘I find myself having a lack of daily routine, before we had a schedule and now, we have the freedom of pacing ourselves, which decreases my motivation.’*

It is possible that the resulting dissatisfaction of UK physiotherapy students was in part due to previous traditional on campus delivery and comparisons made against this norm.

Survey studies completed during the COVID-19 pandemic in India, [45] Pakistan, [46] Libya, [47] Philippines, [48] and Poland [18] have reported that the majority of medical students had a negative perception or expressed dissatisfaction towards online learning. In the UK, conclusions from 2721 medical students across 39 medical schools, suggested students did not find online teaching to be engaging or enjoyable, with limited opportunities to ask questions, and did not find it as effective as face-to-face teaching [44]. Dost et al. [44] reported that 82.17% of students felt that they could not learn practical clinical skills through online teaching with the authors acknowledging that clinical skills remain a pertinent barrier to online teaching. This concern was also expressed by physiotherapy students in our survey, with 94% of respondents feeling disadvantaged by the reduced opportunity to practice ‘hands-on skills,’ and 63% reporting that they were concerned this would affect their future employability. Although further exploration of this is beyond the reach of this study, it would be pertinent to investigate the longer-term impact of the shift to online delivery during the COVID-19 pandemic on the acquisition of clinical skills, student success in examinations and employment.

In the existing literature, students have reported experiencing a lack of motivation, difficulty concentrating, decreased faculty and peer interaction as further limitations of online learning [44]. In continuity with the aforementioned studies, commonly perceived barriers to using online teaching platforms included family distraction (26.76%) and poor internet connection (21.53%). The accumulative effects of these negative perceptions to online learning using metrics such as student attendance and achievement were not investigated in this study. However, in nursing students, investigations have shown that that academic performance was inversely related to the students’ experience, with 43.6% considerably affected and 30.6% greatly affected by the transition to online learning [49]. This outcome may imply that the overall dissatisfaction, anxiety towards online learning and perceived barriers that have been identified, may also have a contributing impact not only on the students experience but also on other critical variables including engagement, achievement and overall success during this period.

Despite respondents’ overall dissatisfaction with online learning within our study, perceived advantages were identified. This method of delivery can be extremely convenient allowing students to engage at times and in locations that are flexible [40]. Just under half the student physiotherapists surveyed agreed that such flexibility is a benefit of an online delivery (49%, n =116, 95% CI 43 to 55). This was especially important during the COVID-19 pandemic where students experienced lockdown rules or periods of isolation. The improved use of technology and digital skills (58%, 137, 52 to 64), ability to learn at one’s own pace and the convenience of home learning (49%, 116, 43 to 55) were all suggested as advantages of online teaching. Some further examples of advantages highlighted by students were evidenced in their qualitative responses.

*‘The increased use of virtual meetings and lecturer catch-ups have increased the accessibility of the teaching team.’*

*‘I like the safety of the home environment as it’s less intimidating compared to large lecture theatres and practical sessions.’*

*‘I am more productive at home with more time for study and research, due to not having to travel to campus.’*

Similar benefits have been identified by UK medical students, with reduced travel time, increased flexibility, the ability to learn at an individual pace, increased comfort and reduced costs associated with online teaching approaches [44].

### Synchronous and asynchronous delivery

A mixture of both synchronous and asynchronous delivery approaches was preferred by respondents. The ability to clarify learning with faculty (87%, 205, 82 to 91) and the structured nature of a synchronous delivery pattern (70%, 165, 64 to 75) was valued by the majority of physiotherapy students surveyed. Synchronous delivery has resulted in overall positive experiences reported by students across sectors including education and technology [50, 51]. Such synchronous delivery can facilitate a stronger feeling of connection to peers and faculty, [52] a perceived disadvantage of online learning identified by physiotherapists in our study. A community of practice is developed through the increased student engagement provided by real-time association between students and academic staff [11, 53]. The benefits of synchronous delivery included a stable platform for student communication, increased task focus, which infuses a greater sense of participation whilst increasing student outcomes [54, 55]. Qualitative themes emerged highlighting the importance of student interaction during synchronous sessions (Table 6). Active learning and engagement are imperative for student learning and the development of collaboration with peers is intrinsically linked to students’ perceptions of engagement [56, 57]. It is important that when the physiotherapy curriculum is delivered online, a range of pedagogical activities are undertaken to engage cohorts, in order to improve satisfaction which is linked to student success.

Asynchronous delivery was also perceived to provide benefits to the physiotherapy curriculum delivery. Respondents found the flexible learning strategy (76%, 179, 70 to 81), where recording of resources could be viewed multiple times (80%, 189, 75 to 85) allowed them to learn at their own pace (74%, 74, 68 to 79). This was highlighted by the qualitative theme ‘pre-reading materials’ (Table 7). The advantages of a flexible approach to learning with a self-paced approach to study has previously been acknowledged [10, 58]. Interaction can still be achieved, e.g., discussion boards, allowing students the opportunities to fully express themselves without the time pressures of live interaction and responding directly to questions in real-time [59]. Two further themes were derived from students responses within our study (Academic input; Learning validation) with several additional suggestions made to enhance interaction both within and following asynchronous sessions (Table 7).

### Academic delivery

Respondents were ‘impartial’ (3.13 ± 1.18) when asked if they felt supported by the academic faculty during online learning sessions. Importantly, just under half of the respondents (45%) felt that the faculty had the necessary skills to deliver effective online content meeting the curriculum outcomes. A wide range of teaching strategies were experienced, similar to the variety a traditional on campus delivery would include, however respondents acknowledged inconsistency in academics technological proficiency or confidence to teach online.

*‘Some lecturers need additional support to improve their technical literacy with new features and ensure they have the support necessary to engage with and deliver these online classes’.*

*‘Most staff are very technologically proficient or can figure out a solution to a problem quickly. However, one member of staff is so confused by technology it is cringeworthy and my peers and I dread any interaction with them online.’*

At the start of the lockdown period within the UK, academics were asked to transition contemporary teaching online in what has been described as ‘emergency remote teaching’ [60, 61]. The lack of preparation and planning time to deliver a curriculum which was not intended for an online delivery method may explain why respondents felt some lecturers lacked the necessary skills to provide effective online education. Faculty members new to online learning take time to understand their different roles and responsibilities in the new modality of learning and teaching [35, 62]. It is the pedagogy and not technology which is critical to the success of online delivery [63–65]. Staff having the time to plan and organise effective learning strategies, embracing new skills to reach distance learners, will provide a more enriching student experience [35, 38]. Understanding lecturers’ perceptions of online learning within healthcare would be an important step to develop competence in the area.

A limitation of this study includes the possibility of responder/non-response bias [66]. It is possible that students who responded to the questionnaire are not representative of the entire target population [67]. Whilst the approach undertaken was applied to capture students’ overall perceptions of physiotherapy online learning in higher education, further research is required to understand the reasons for respondents’ answers. These should be measured against important student outcomes in terms of clinical proficiency, achievement and employability. Future research should also explore how the academic faculty perceived the shift from traditional face-to-face campus delivery to a hybrid or purely online delivery model. This would provide a more complete picture of key stakeholders in higher education. With the emergence of mainstream telehealth, a solution to many access barriers and a viable and effective alternative for those who cannot access mainstream physiotherapy, [68] it is imperative that physiotherapy students are digitally competent. As higher education institutions review on-going provision to ensure that students are prepared for the challenges of the healthcare system, [8] the adoption of online learning and the advantages of a hybrid delivery model, need to be implemented with consideration to the challenges described in this survey.

Furthermore, as students become familiar with this pedagogical approach, additional exploration would provide a clearer perspective of attitudes towards online learning. Future investigation of key constructs identified from this exploratory study would help to provide clarity of how the pedagogical landscape has evolved. ‘Student engagement’ (e.g. attitudes towards online learning), ‘cohort identity’ (e.g. peer interaction and support), ‘standards of delivery’ (e.g. digital platforms, staff IT competency) and ‘barriers to learning’ (e.g. connectivity, family responsibilities, digital literacy) were all identified as key constructs from this investigation. Evaluation of these constructs would help to establish how this pedagogical approach has developed in physiotherapy education and if adherence to online learning is valued by future cohorts as we emerge from the pandemic.

## Conclusion

The UK physiotherapy students who completed this study felt that the online learning delivery had a negative impact on their understanding of the subject and were disadvantaged compared to face-to-face traditional provision. Several advantages and disadvantages to both synchronous and asynchronous delivery were highlighted. Physiotherapy content delivered during online classes require pedagogical strategies that will create as many learning and engagement opportunities as possible. Just under half the respondents believed that academic faculty lacked the necessary skills to deliver effective online content. Future research may wish to explore the impact of online course delivery from both academic faculty and student perspectives focusing on constructs identified in this study. These constructs should be considered in relation to outcome metrics including completion rates, attainment, clinical proficiency and graduate employment.

## Supplementary Information

**Additional file 1.**

## Data Availability

The datasets generated and analyzed during the current study are not publicly available due to limitations of ethical approval involving the participant data and anonymity but are available from the corresponding author on reasonable request.

## Abbreviations

BSC: Bachelor of Science
MSC: Master of Science
UK: United Kingdom
WHO: World Health Organisation
