# Supplementary material for: Student physiotherapists perceptions of online curriculum delivery during the COVID-19 pandemic
Source: BMC Med Educ. 2022 Jun 7;22:440. doi: 10.1186/s12909-022-03486-5 (PMC9172598; doi:10.1186/s12909-022-03486-5)
Supplement: Supplementary file 1 — Additional file 1. [file 12909_2022_3486_MOESM1_ESM.docx]

**Supplementary Material 1 - Survey Questions**

**Respondent Characteristics**

Section 1: Physiotherapist students background Characteristics

1.Please state your age

2.How do you identify?

3. Please state your ethnicity

4. Which type of degree are you registered on?

5. Please confirm your year of study

Section 2: Online Learning Perceptions

6. How much do you agree with the following: I feel at a disadvantage with online compared to face to face learning (Likert scale – 5 – Strong Agree, 4- Agree, 3 - Impartial, 2- Disagree, 1 – Strongly Disagree)

7. How have you found the transition to online learning from face-to-face delivery? Apprehensive about change

Not worried

Can see the benefits/opportunities of using technology

Other

8. What do you believe are the advantages of online learning? (tick all those that apply

Improve use of technology and digital skills for future use

Interact with groups of students virtually

More confident to interact/answer questions (secondary to anonymity)

Find digital quizzes useful for learning

Find video’s/demonstrations useful to supplement learning

Able to learn at own pace better (secondary to recorded sessions)

The convenience of learning in home environment

Other

I don't believe there are any advantages to Online learning

9. What do you believe are the disadvantages of online learning? (tick all those that apply Lack of cohort identify

Unable to develop close relationships with peers

Lack of peer feedback during sessions

Lack of one-to-one feedback from tutors

Unable to practice ‘hands on’ skills

Decrease chances of employability – if lack exposure to clinical skills

Lack of confidence in using the technology

Connectivity issues during sessions

Pace of delivery can be affected

Doesn’t meet preferred learning style

Lack of designated workspace or study area (either at University or personal residence)

Other

I don't believe there are any disadvantages to Online learning

10. Has online learning had a negative impact on your understanding of the subject area?

11. If Yes, please give reasons for your answer:

Pace of delivery affected my understanding

Connectivity issues – missed aspects of sessions

Lack of confidence in applying clinical/practical skills following online delivery

Not confident using the technology which then affects my ability to concentrate on acquiring knowledge of the subject area

Other

12. If No, please give reasons for your answer

Recorded sessions have allowed for better understanding

Technology used/Apps have been useful

I feel that my learning is similar or superior to a face-to-face delivery format

The advantages of online learning outweigh the disadvantages

Other

13. Do you prefer synchronous (online 'live' delivery) or asynchronous learning (pre-recorded sessions to view at your own pace)?

14. What are the benefits of synchronous learning? (Tick all that apply)

Can clarify points with tutors

More interactive with peers

Structured learning (makes you attend the session)More engaged with subject material

More motivated to learn

I don't believe there are benefits to synchronous learning

Other

15. What are the benefits of asynchronous learning?

Do at your own pace

Watch recordings as many times as you wish

Flexible learning (I.e. at a time of your choice)Improves work/life balance

I don't believe there are benefits to asynchronous learning

Other

16. Do you agree with the following statement? I feel equally motivated to learn using an online learning compared to a face to face approach (Likert scale – 5 – Strong Agree, 4- Agree, 3 - Impartial, 2- Disagree, 1 – Strongly Disagree)

17. Do you agree with the following statement? I am satisfied with the online learning approach within my degree programme. Explain your answer(Likert scale – 5 – Strong Agree, 4- Agree, 3 - Impartial, 2- Disagree, 1 – Strongly Disagree)

18. Do you agree with the following statement? I have felt supported by my tutors for online learning sessions Explain your answer(Likert scale – 5 – Strong Agree, 4- Agree, 3 - Impartial, 2- Disagree, 1 – Strongly Disagree)

19. What further support do you feel is required to make online learning most effective for students?

Further advice on how to use the technology

Training in technology for students (outside of taught sessions)

Further explanation of the value of it

Technology Hardware support (purchasing/loaning equipment/including data and wifi)

Areas provided by the University to study 'online' in appropriate environments (e.g. library spaces)

Other

20. How do you interact (engage) within online classes?

Same as FTF classes

The opportunities are available, but I chose not to online

The opportunities to engage are not the same as FTF classes

I engage more with online delivery

21. Is there anything you would like to be included in synchronous (live sessions) sessions that would be helpful with your learning?

22. Is there anything you would like to be included in the asynchronous (pre-recorded) sessions that would be helpful with your learning?

23. Do you think academic staff/lecturers have the necessary skills to deliver effective online training?
